# Backward walking training improves balance in school-aged boys

**DOI:** 10.1186/1758-2555-3-24

**Published:** 2011-10-22

**Authors:** Wei-Ya Hao, Yan Chen

**Affiliations:** 1 China Institute of Sport Science, Beijing, China; 2 Guangdong Provincial Institute of Sport Science, Guangzhou, China

**Keywords:** Motor control, Kinematics, Gait, Backward walking, Male children

## Abstract

**Background:**

Falls remain a major cause of childhood morbidity and mortality. It is suggested that backward walking (BW) may offer some benefits especially in balance and motor control ability beyond those experienced through forward walking (FW), and may be a potential intervention for prevention of falls. The objective of this study was to investigate the effects of BW on balance in boys.

**Methods:**

Sixteen healthy boys (age: 7.19 ± 0.40 y) were randomly assigned to either an experimental or a control group. The experimental group participated in a BW training program (12-week, 2 times weekly, and 25-min each time) but not the control group. Both groups had five dynamic balance assessments with a Biodex Stability System (anterior/posterior, medial/lateral, and overall balance index) before, during and after the training (week- 0, 4, 8, 12, 24). Six control and six experimental boys participated in a study comparing kinematics of lower limbs between FW and BW after the training (week-12).

**Results:**

The balance of experimental group was better than that of control group after 8 weeks of training (*P *< 0.01), and was still better than that of control group (*P *< 0.05), when the BW training program had finished for 12 weeks. The kinematic analysis indicated that there was no difference between control and experimental groups in the kinematics of both FW and BW gaits after the BW training (*P *> 0.05). Compared to FW, the duration of stance phase of BW tended to be longer, while the swing phase, stride length, walking speed, and moving ranges of the thigh, calf and foot of BW decreased (*P *< 0.01).

**Conclusion:**

Backward walking training in school-aged boys can improve balance.

## Introduction

Falls remain a major cause of childhood morbidity and mortality, and it is the leading cause of unintentional injuries among children causing between 25% and 44% of injuries [1-3]. Moreover, falls among elderly people is a serious medical and societal challenge. Between 25% and 35% of people older than 65 suffer from one or more falls every year [4]. Injuries caused by falls not only affect elderly people, who are frail or impaired, but young healthy individuals as well [5,6]. Many factors such as decline of balance and lean body mass, decrease of muscular strength of lower limbs and weakening of visual, cutaneous and proprioceptive, and vestibular senses and etc may lead to falls [7,8]. Balance, which issues from the interaction of the sensory system, the motor system and the musculoskeletal system, plays a great role among all the factors [7,8].

In order to prevent the occurrence of falls, exercise is often introduced to increase the ability of balance and motor control. Tai Chi Chuan (TCC) [9,10] and Tae Kwon Do (TKD) [11] are among those coordination exercise that have been thought to be suitable for older people and adult patients with chronic disease. There are some studies about exercises promoting balance ability in children with Down syndrome [12], intellectual disabilities [13], coordination disorder [14], and obese and inactive children [15]. However, to our knowledge, there is no study regarding effects of exercise upon ability of balance and motor control for healthy children. All exercises used in those studies for adults or children are not easy to learn and practice, especially for children. Simpler physical exercise, such as backward walking (BW) exercise may have same beneficial effect for healthy children, especially in balance and motor control ability [16,17]. In this paper, we test the hypothesis that BW could improve balance in school-aged boys. We also test detraining effects on balance after stopping the exercise. Furthermore, we compare spatiotemporal gait characteristics after BW training, and investigate differences of spatiotemporal gait characteristics between normal forward walking (FW) and BW, which may contribute to the improvement of balance, if any.

## Methods

Sixteen healthy boys without any motor disorders (age: 7.19 ± 0.40 y) were recruited to this study. After parents and boys agreed to participate in the study, they signed written informed consent, which was in compliance with the Helsinki Declaration and internationally recognized guidelines. The study was approved by the local institutional ethics committee (China Institute of Sport Science, China).

The boys were randomly assigned to the control and experimental groups. There was no difference between the control and experimental groups in age (7.25 ± 0.46 vs 7.13 ± 0.35 y), body mass (32.56 ± 6.86 vs 29.26 ± 4.67 kg) and height (129.49 ± 5.63 vs 128.19 ± 7.75 cm).

Participants of the experimental group had finished a 12-week BW training program. The training sessions were 2 times weekly, 25 min each time, and implemented into physical education classes in a 60 m long straight track in a playground. The gait speed and pace were not imposed. There was no constraint or indication about head and trunk position during BW training. During the BW training of the experimental group, the control group took their normal physical exercises, directed by a PE teacher and comprised various activities and games focusing on balance, strength, endurance, and courage and on having fun. The intensities of exercise were same between groups. The participants had their normal school life except the physical education class.

Dynamic postural stability, which emphasized a subject's ability to maintain center of balance, was assessed using a Biodex Balance System (BSS) (Biodex, Shirley, New York, USA). BSS has a circular platform that is free to move about the anterior-posterior (AP) and medial-lateral (ML) axes simultaneously [18,19]. The BSS software sampled the deviations in the AP and ML directions at a rate of 20 Hz and calculated the anterior/posterior index (API), medial/lateral index (MLI), and overall balance index (OBI) using the following formulas. It is reported that these indexes are reliable and precise measures for dynamic postural stability [19,20]. The intra-tester reliability of BBS were 0.82 [19] and 0.96 [20], and the inter-tester reliability was 0.70 [19] for OBI.

$$API=\sqrt{\frac{\sum {\left(0-Y\right)}^{2}}{N}}$$

$$MLI=\sqrt{\frac{\sum {\left(0-X\right)}^{2}}{N}}$$

$$OBI=\sqrt{\frac{\sum {\left(0-Y\right)}^{2}+\sum {\left(0-X\right)}^{2}}{N}}$$

where N is the number of sampling, Y and X are displacement of COP in the AP and ML directions. Thus, OBI, API and MLI represent the subjects' ability to control their balance in all directions, in the sagittal plane, and in the frontal plane respectively [18,19].

The BBS allows for varying levels of difficulty of stability testing, ranging from level 8 (most stable) to level 1 (least stable). Five balance assessments were conducted for both the experimental and control groups. The first assessment was conducted in the week prior to initiation of the BW training. The second, third, and fourth were after the 4^th^, 8^th ^and 12^th ^week. The last was after 12 weeks of stopping the BW training. The balance tests were conducted complying with the procedures for testing postural control [18,19]. The difficulties of stability level were increased progressively in the five assessments (Table 1).

**Table 1:** Stability levels in the balance assessments

	Starting level	Ending level
week 0	8	8
week 4	8	5
week 8	6	4
week 12	4	4
week 24	4	4

Notice: The movable balance platform was the most stable at level 8, while was the least stable at level 1. The stabilities were equably decreased from starting level to ending level during 20 s testing session, in the balance assessments of week 4 or 8.

After the 12-week BW training of the experimental group, 6 control and 6 experimental boys participated in the kinematical study. After the subjects were familiar to the trial, kinematical data were obtained at 100 Hz using a Qualisys Motion Capture System (Gothenburg, Sweden) with 6 cameras mounted around the 12 m long walkway in a laboratory. A calibration frame of the Qualisys system was used for calibration the space before the kinematic test. The test-to-test reliability was almost absolute (r = 0.99) and the position-to-position reliability was good (r = 0.88). The positions of selected points on both left and right side of the lower limbs were recorded by attaching 10 spherical reflective markers (15 mm in diameter) to the skin overlying the following bony landmarks: anterior superior iliac spine, greater trochanter, a point midway between the lateral epicondyle of the femur and the fibula head, lateral malleolus, and second metatarso-phalangeal joint on the superior aspect of the foot. A 2D model in sagittal plan was used with 10 markers defining angles of hip, knee and ankle.

The boys were instructed to walk at a self-selected speed, look straight ahead, and walk as naturally as possible. Each child had finished 3 times of BW trial and 3 times of FW trials. Kinematics of the lower limbs in sagittal plane, including, stride lengths and rates, angle ranges, and stance and swing durations of the stride cycle, were extracted for statistical analysis. The walking data of the 3 times had been averaged after being calculated from each trial and analyzed. A whole stride cycle here refers to the phase from one foot contact to the next contact of the same foot, a step refers to the phase from one foot contact to the next contact of the other foot, and a stride length refers to the distance covered by a foot from one contact point to next contact.

Software SPSS 13.0 was employed for statistic analysis. The parameters were firstly tested using Normal Q-Q plot for normal distribution in order to further statistic analysis. Independent-Samples T Test was used to compare the corresponding balance indexes between experimental and control groups (*P *< 0.05). When significance found, Cohen's effect size (ES) was used to measure the magnitude of the BW training effect [21]. Two-way analysis of variance (ANOVA) tests, followed by *post hoc *Tukey test, were performed to detect differences in kinematic variables between control and experimental groups (between-subject), and gait differences between FW and BW (within-subject). A significance level of 0.05 was chosen for all the statistical analysis.

## Results

**Figure 1:**
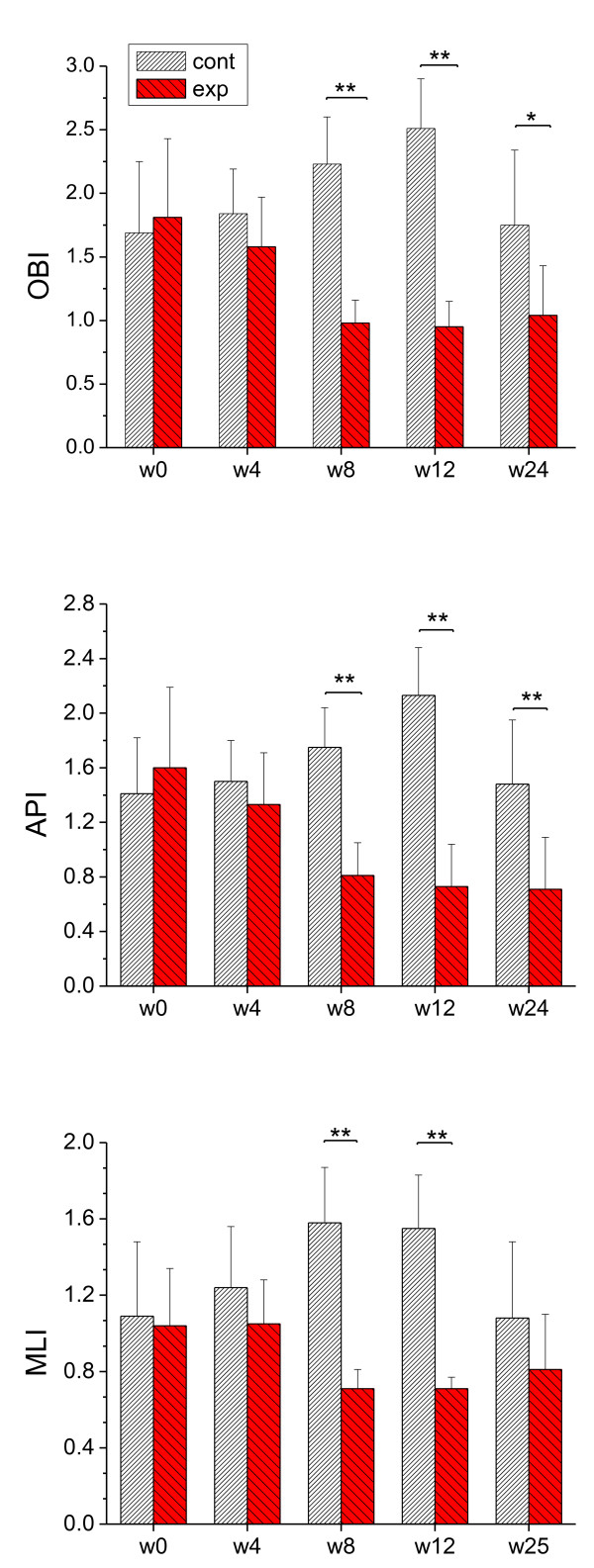
**Dynamic balance indexes of the subjects in control (cont) and experimental (exp) groups**. OBI: overall balance index; API: anterior/posterior index; MLI: medial/lateral index. Lower values indicate better balance. The difficult levels of the balance tasks for the five tests (week 0, week 4, week 8, week 12, week 24) were different, thus they were not comparable among the five tests. *P < 0.05, **P < 0.01.

As shown in Figure 1, after 8 weeks BW training, the three balance indexes of the experimental group were all significantly less than those of control group (*P *< 0.001; ES = 3.56~5.07). Moreover, 12 weeks after finishing the BW training program, there were still significant differences in OBI (*P *= 0.012; ES = 1.44) and API (*P *= 0.003; ES = 1.77), although there was no significant difference (*P *> 0.05) in MLI, between experimental and control groups.

Two-way analysis of variance tests showed no interaction effect in kinematic variables between factors of groups (control and experimental) and gait differences (FW and BW) (P > 0.05). It also indicated that there was no difference in the kinematic variables between the experimental and control groups (*P *> 0.05). By contrast, there were many differences in the kinematic variables between BW and FW gaits. Comparing to FW, relative time spent in the double foot support phase was increased even though the double foot support phase in FW was already very long, but the swing phase was decreased (Table 2). The long double support phases suggest that the boys might be prudent during the test. Moreover, the stride lengths, walking speed of BW were less than those of FW, although gait cycles of the two kinds of ambulation were very close (Table 3). From these comparisons, it is suggested that (1) there was no difference between control and experimental groups in the kinematics of both FW and BW gaits, (2) there are some gait differences in BW from normal FW gait.

**Table 2:** Proportions of stance and swing duration in gait cycles during forward and backward walking

	Left		Right	
	
	Forward	Backward	Forward	Backward
Stance phase	0.69 (0.07)	0.71 (0.05)	0.64 (0.05)	0.74 (0.06)**
Single support	0.36 (0.05)	0.25 (0.05)**	0.32 (0.06)	0.27 (0.07)
Double support	0.33 (0.09)	0.46 (0.07)**	0.32 (0.09)	0.47 (0.10)**
Swing phase	0.32 (0.07)	0.29 (0.05)	0.36 (0.05)	0.26 (0.06)**

* *P *< 0.05, ** *P *< 0.01, vs Forward.

**Table 3:** Comparison of gait parameters between forward and backward walking

	Forward	Backward
Step length left (mm)	423.76 (60.91)	317.72 (79.87)**
Step length right (mm)	487.22 (49.26)	298.75 (102.58)**
Stride length (mm)	935.77 (161.48)	634.74 (159.64)**
Stride cycle (s)	0.92 (0.09)	0.96 (0.14)
Speed (mm/s)	1055.85 (173.60)	694.91 (123.32)**
Step length left×10/height	3.27 (0.40)	2.46 (0.65)**
Step length right×10/height	3.76 (0.37)	2.30 (0.75)**

* *P <*0.05, ** *P *< 0.01, vs Forward.

There were significant differences in angles at hip, knee, or ankle between FW and BW (Table 4). When the walking direction reversed to backwards, all changing ranges of joint angles at lower limb tended to shift and decrease, maximal extensions of hip and flexions of knee were decreased as well. In BW gait, there were about 11° maximal dorsiflexion with feet, but there were almost no dorsiflexion in FW gait. The maximal ankle angles of BW were about 16° less than those of FW indicating that the feet had less plantar flexion in BW.

**Table 4:** Angle ranges of lower limbs during forward and backward walking

		Left		Right	
		
		Forward	Backward	Forward	Backward
Hip	Minimum	114.03 (13.09)	113.52 (9.65)	119.25 (15.03)	115.89 (15.83)*
	Maximum	139.79 (8.99)	132.97 (7.66)**	144.03 (13.93)	137.22(14.83)**
	Range	25.76 (5.33)	19.45 (3.31)**	24.77 (5.53)	21.33 (6.15)
					
Knee	Minimum	114.14 (8.70)	123.53(11.07)**	112.99 (7.00)	121.07(11.01)**
	Maximum	173.05 (6.27)	171.78 (4.59)	173.53 (4.78)	173.70 (4.15)
	Range	58.91 (9.15)	48.25 (9.19)**	60.54 (7.01)	52.63 (8.87)*
					
Ankle	Minimum	91.10 (11.99)	81.92 (6.49)**	90.54 (10.87)	78.45 (5.14)**
	Maximum	123.01 (9.24)	109.14(10.26)**	121.77 (9.99)	104.26 (3.56)**
	Range	31.91 (8.24)	27.22 (6.41)	31.23 (8.63)	25.80 (5.16)*

* *P <*0.05, ** *P *< 0.01, vs Forward.

## Discussion

The results of this study indicated that the balance of the boys had been significantly improved after 8 weeks of BW training (Figure 1). The balance ability, especially in the anterior/posterior direction, was still maintained at a higher level, when the BW training program had stopped for 3 month. It is considered that the effect is obvious when ES is greater than 0.8 [21]. In this study, the ES values aforementioned were all greater than 1.4 suggesting that the training effect was extremely large. Our results are similar to findings by Zhang et al. [17] and Yang et al. [22]. Zhang et al. [17] measured and compared static balance abilities of 18 old women (experimental group) before and after 12 weeks BW training with that of 12 old women (control group) without BW training using a force plate and an electronic apparatus for single standing test. They found that the single leg standing duration of the experimental group was increased and greater than that of control group; the fluctuation of gravity center of static standing with eye closed of the experimental group was decreased and less than that of control group [17]. Yang et al. [22] measured gait patterns of two groups (control, n = 12; experimental, n = 13) of patients post stroke using a gait analysis system, and found that after a three-week BW training period, subjects of experimental group showed more improvement than those in control group for walking speed, stride length, and symmetry index.

The exact mechanism through which BW exercise cause improvement of balance and motor control is yet to be fully elucidated. It has been generally assumed that there are many systems within the body that work in concert to move the center of mass (COM) in relation to the base of support (BOS) in a controlled manner when engaged in dynamic tasks [23]. There are three primary systems involved for the balancing process: (1) the sensory system (visual, cutaneous and proprioceptive, and vestibular senses), which gives feedback to alter the balance action during a voluntary motor task, (2) the motor system, which creates the coordination movement to maintain balance, and (3) the biomechanical system or musculoskeletal system, which includes the muscles that create the movement torques and the bony and joint frame on which movements are made [23].

All those three systems may be associated with the improvement of balance by BW exercise. Children rely more on visual cues than the other sensory cues [24], but children can reweight the three afferent cues since 3 years old in order to maintain balance, and this multisensory reweighting increases with age in children [25]. During BW, the visual cues doesn't provide the child with the visual information necessary to anticipant ground condition, and motor pattern are unconventional, the boys have to reorganize and adapt the changed information from visual, cutaneous and proprioceptive, and vestibular senses, and then enhance the movement control to maintain dynamic balance [26]. It has been reported that prolonged BW exercise causes neural adaptations. Schneider and Capaday [27] and Ung et al. [28] found that daily BW training progressive induced adaptation of the soleus H-reflex. Van Deursen et al. [29] suggested that both FW and BW are mediated by the same central pattern generator (CPG), and only small modifications in the CPG are required in order to produce the different characteristics of each walking mode. The reorganization of the muscle synergies or neuromotor control in lower limbs during BW might be a possible reason for the improvement of balance by BW exercise.

The differences of BW gait patterns from FW may also play roles in the mechanism of BW exercise causing improvement of balance. Stance begins with heel strike and ends at toe-off in FW. On the contrary, in BW, the toes contact the ground first and the heel is lifted of the ground at the end. Winter et al. [30] suggested that BW was a near image of FW, and suggested that in order to produce the muscle activation patterns involved in FW the temporal cycling of the muscle contractions in BW was simply reversed. Grasso et al. [31] found that the waveforms of all elevation angles in BW gait were essentially time reversed relative to the corresponding waveforms in FW gait. However, our results indicated that the kinematics of BW was somewhat different than those of FW. It seems that the boys were more prudent during BW and spent more time on double support phase (Table 2). The gait speed and stride length of BW were less than those of FW, although step duration was found not to significantly differ between BW and FW conditions (Table 2 and 3). Moreover, the ranges of motion (ROM) of thigh, calf and foot of FW were reduced, and shifted in foot, compared with FW (Table 4). Such shifting and reduction of lower limbs ROM had been found in previous findings [31,32]. Nevertheless, one result, which the durations of stance and swing phases maintained different proportions of stride cycles in BW and FW in this study, contradicts to the findings [31,32]. Further investigation on the difference in walking cadence of BW from FW gait would provide more insights into the reorganization of the relative phase pattern.

The changes of muscles strength at lower limbs may contribute to the improvement of balance induced by BW exercise as well. The contraction modes of lower limb muscles are reversed in BW conditions. For example, eccentric contraction of the quadriceps muscle during the loading phase of the FW gait is replaced by a concentric contraction during BW [33]. Previous studies indicate that strengths of quadriceps and hamstring muscles are increased after BW exercise [34-36].

Although we found that BW increased balance ability for boys as those old women [17] and patients [22], we still don't know whether the mechanism involved in these boys was same as those old women or patients [17,22]. The three sensory components of sensormotor control system develop in different periods [37]. Proprioceptive function seemed to mature at 3 to 4 years of age, but visual and vestibular afferent systems reached adult level at 15 to 16 years of age [37]. The boys in this study were only at 7 to 8 years of age.

As aforementioned, compared with other physical exercises for improving balance, such as TCC [9,10], TKD [11], or Square-stepping exercise [38], BW is much easier to put into practice. BW also offer benefits especially in balance and motor control ability beyond those experienced through FW alone. In comparing with FW, BW results in the mean electromyographic (EMG) activity of the lower extremities over the gait cycle [31,32], which suggests a greater level of energy expenditure during BW than FW. In fact, it has been found that, during BW, oxygen consumption and heart rate are much greater than during matched speed FW [36,39], suggesting that BW need more metabolic cost, and provide more stimulus to maintain fitness of cardiovascular system. Due to its improvement of motor control ability [17,22] and reducing impact upon knee joints [40], backward ambulation can be used as a rehabilitation technique for treating patients post stroke [22] and with orthopeadic problems, especially those involving knee dysfunction [16,34].

## Conclusion

This study indicates that BW training on school-aged boys can improve their balance. It has found that there was no difference between control and experimental groups in the kinematics of both FW and BW gaits after the BW training. It also indicates that the spatiotemporal variables such as durations of stance and swing phase, stride length, walking speed, and ranges of angular displacements of the lower limbs of BW are different from those of FW. These differences may contribute to the improvement of balance induced by BW training. These findings may provide useful information to promote balance ability in prevention of fall injuries through BW training, and promote physically active lifestyle. They may also provide useful information to understand the mechanism of improvement of balance induced by BW training.

## Competing interests

The authors declare that they have no competing interests.

## Authors' contributions

Both authors conceived of the study, determined the design, performed the statistical analysis, interpreted the data and prepared the manuscript. They have read and approved the final manuscript.
